# 2-Nitro-*N*-propyl­benzamide

**DOI:** 10.1107/S1600536809020728

**Published:** 2009-06-06

**Authors:** Li-Hua Guo, Hai-Jun Tan, Ji-Kui Wang, Jin-Tang Wang

**Affiliations:** a Department of Applied Chemistry, College of Science, Nanjing University of Technology, Nanjing 210009, People’s Republic of China

## Abstract

The title compound, C_10_H_12_N_2_O_3_, contains three mol­ecules in the asymmetric unit. In the crystal structure, inter­molecular N—H⋯O inter­actions link the mol­ecules into chains along the *b* axis. The crystal structure is consolidated by weak C—H⋯π inter­actions.

## Related literature

The title compound is an agent for treating and preventing pains, see: Goodman & Serafini (2004 ▶). For bond-length data, see: Allen *et al.* (1987 ▶).
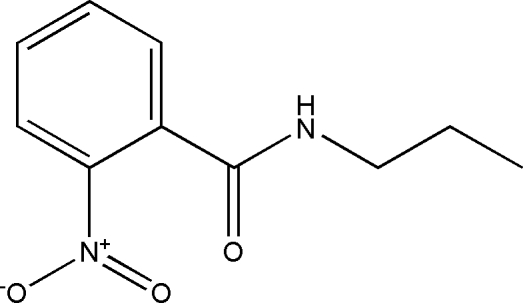


## Experimental

### 

#### Crystal data

C_10_H_12_N_2_O_3_
*M* *_r_* = 208.22
Monoclinic, 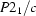

*a* = 15.835 (3) Å
*b* = 9.3910 (19) Å
*c* = 23.618 (5) Å
β = 108.35 (3)°
*V* = 3333.6 (12) Å^3^
*Z* = 12
Mo *K*α radiation
μ = 0.09 mm^−1^
*T* = 298 K
0.30 × 0.20 × 0.10 mm

#### Data collection

Enraf–Nonius CAD-4 diffractometer
Absorption correction: ψ scan (North *et al.*, 1968 ▶) *T*_min_ = 0.973, *T*_max_ = 0.991
6289 measured reflections
6056 independent reflections
2855 reflections with *I* > 2σ(*I*)
*R*_int_ = 0.060
3 standard reflections every 200 reflections intensity decay: 1%

#### Refinement

*R*[*F* ^2^ > 2σ(*F* ^2^)] = 0.079
*wR*(*F* ^2^) = 0.188
*S* = 1.00
6056 reflections
388 parameters
H-atom parameters constrained
Δρ_max_ = 0.29 e Å^−3^
Δρ_min_ = −0.56 e Å^−3^

### 

Data collection: *CAD-4 Software* (Enraf–Nonius, 1985 ▶); cell refinement: *CAD-4 Software*; data reduction: *XCAD4* (Harms & Wocadlo, 1995 ▶); program(s) used to solve structure: *SHELXS97* (Sheldrick, 2008 ▶); program(s) used to refine structure: *SHELXL97* (Sheldrick, 2008 ▶); molecular graphics: *SHELXTL* (Sheldrick, 2008 ▶); software used to prepare material for publication: *SHELXTL*.

## Supplementary Material

Crystal structure: contains datablocks I, Il. DOI: 10.1107/S1600536809020728/at2796sup1.cif

Structure factors: contains datablocks I. DOI: 10.1107/S1600536809020728/at2796Isup2.hkl

Additional supplementary materials:  crystallographic information; 3D view; checkCIF report

## Figures and Tables

**Table 1:** Hydrogen-bond geometry (Å, °)

*D*—H⋯*A*	*D*—H	H⋯*A*	*D*⋯*A*	*D*—H⋯*A*
N1—H1*A*⋯O4^i^	0.86	2.02	2.854 (5)	163
N3—H3*C*⋯O1	0.86	1.98	2.840 (4)	177
N5—H5*A*⋯O7^ii^	0.86	2.04	2.843 (4)	154
C6—H6*A*⋯*Cg*2	0.93	2.86	3.751 (5)	162

Symmetry codes: (i) 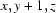; (ii) 
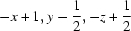
. *Cg*2 is the centroid of the C15–C20 ring.

**Fig. 1.**
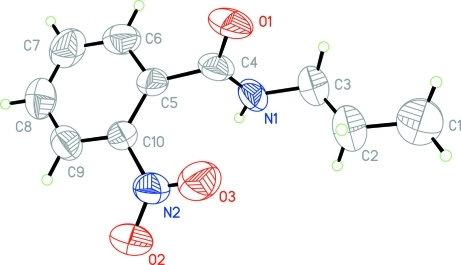
The one molecule of the three independent molecules in asymmetric unit, with the atom-numbering scheme. Displacement ellipsoids are drawn at the 50% probability level.

**Fig. 2.**
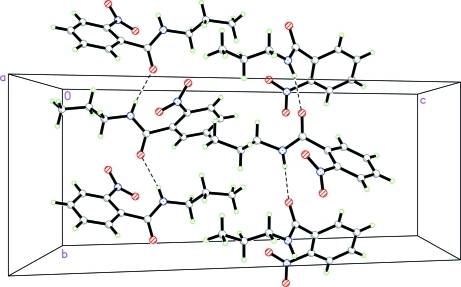
A packing diagram for (I).

## supplementary crystallographic information

### Comment

The title compound is a kind of medicament for treating and preventing pains, and traumatic injuries such as traumatic brain injury and acute spinal cord injury (Goodman & Serafini, 2004). We herein report the crystal structure of the title compound (I).

In the molecule of (I), (Fig. 1), the bond lengths (Allen *et al.*, 1987) and angles are within normal ranges. The title compound crystallized in the monolinic space group P2_1_/c, with three independent molecules (A, B and C) in the asymmetric unit.

In the crystal structure, intermolecular N—H···O interactions (Table 1) link the molecules into chains along the *b* axis (Fig. 2), in which they may be effective in the stabilization of the structure. The crystal structure is consolidated by C—H···π hydrogen-bonding interactions (Table 1).

### Experimental

2-Nitro-*N*-propylbenzamide were dissolved in DMF (50 mL). The solution was then poured to ice water. The crystalline product was isolated by filtration, washed with water (600 ml), dried and give the product 1.8 g. The crystals of (I) were obtained by evaporating the acetone slowly at room temperature for about 14 d.

### Refinement

H atoms were positioned geometrically, with N—H = 0.86 and C—H = 0.93-0.97 Å, and constrained to ride on their parent atoms, with *U*_iso_(H) = 1.2 or 1.5*U*_eq_(C,N).

### Crystal data

**Table:**

C_10_H_12_N_2_O_3_	*F*(000) = 1320
*M**_r_* = 208.22	*D*_x_ = 1.245 Mg m^−^^3^
Monoclinic, *P*2_1_/*c*	Melting point: 340 K
Hall symbol: -P 2ybc	Mo *K*α radiation, λ = 0.71073 Å
*a* = 15.835 (3) Å	Cell parameters from 25 reflections
*b* = 9.3910 (19) Å	θ = 9–12°
*c* = 23.618 (5) Å	µ = 0.09 mm^−^^1^
β = 108.35 (3)°	*T* = 298 K
*V* = 3333.6 (12) Å^3^	Needle, colourless
*Z* = 12	0.30 × 0.20 × 0.10 mm

### Data collection

**Table:**

Enraf–Nonius CAD-4 diffractometer	2855 reflections with *I* > 2σ(*I*)
Radiation source: fine-focus sealed tube	*R*_int_ = 0.060
graphite	θ_max_ = 25.3°, θ_min_ = 1.4°
ω/2θ scans	*h* = 0→19
Absorption correction: ψ scan (North *et al.*, 1968)	*k* = 0→11
*T*_min_ = 0.973, *T*_max_ = 0.991	*l* = −28→26
6289 measured reflections	3 standard reflections every 200 reflections
6056 independent reflections	intensity decay: 1%

### Refinement

**Table:**

Refinement on *F*^2^	Primary atom site location: structure-invariant direct methods
Least-squares matrix: full	Secondary atom site location: difference Fourier map
*R*[*F*^2^ > 2σ(*F*^2^)] = 0.079	Hydrogen site location: inferred from neighbouring sites
*wR*(*F*^2^) = 0.188	H-atom parameters constrained
*S* = 1.00	*w* = 1/[σ^2^(*F*_o_^2^) + (0.050*P*)^2^ + 3.*P*] where *P* = (*F*_o_^2^ + 2*F*_c_^2^)/3
6056 reflections	(Δ/σ)_max_ < 0.001
388 parameters	Δρ_max_ = 0.29 e Å^−^^3^
0 restraints	Δρ_min_ = −0.56 e Å^−^^3^

### Special details

**Table:**

Experimental. Refinement of F^2^ against ALL reflections. The weighted *R*-factor *wR* and goodness of fit S are based on F^2^, conventional *R*-factors *R* are based on F, with F set to zero for negative F^2^. The threshold expression of F^2^ > 2sigma(F^2^) is used only for calculating *R*-factors(gt) *etc*. and is not relevant to the choice of reflections for refinement. *R*-factors based on F^2^ are statistically about twice as large as those based on F, and R– factors based on ALL data will be even larger.
Geometry. All e.s.d.'s (except the e.s.d. in the dihedral angle between two l.s. planes) are estimated using the full covariance matrix. The cell e.s.d.'s are taken into account individually in the estimation of e.s.d.'s in distances, angles and torsion angles; correlations between e.s.d.'s in cell parameters are only used when they are defined by crystal symmetry. An approximate (isotropic) treatment of cell e.s.d.'s is used for estimating e.s.d.'s involving l.s. planes.
Refinement. Refinement of *F*^2^ against ALL reflections. The weighted *R*-factor *wR* and goodness of fit *S* are based on *F*^2^, conventional *R*-factors *R* are based on *F*, with *F* set to zero for negative *F*^2^. The threshold expression of *F*^2^ > σ(*F*^2^) is used only for calculating *R*-factors(gt) *etc*. and is not relevant to the choice of reflections for refinement. *R*-factors based on *F*^2^ are statistically about twice as large as those based on *F*, and *R*- factors based on ALL data will be even larger.

### Fractional atomic coordinates and isotropic or equivalent isotropic displacement parameters (Å^2^)

**Table:**

	*x*	*y*	*z*	*U*_iso_*/*U*_eq_	
O1	0.2163 (2)	0.1794 (3)	−0.09514 (14)	0.0812 (10)	
O2	0.3600 (3)	0.6643 (4)	−0.09104 (16)	0.1093 (13)	
O3	0.3303 (2)	0.4769 (4)	−0.04864 (14)	0.0799 (9)	
N1	0.1426 (2)	0.3839 (4)	−0.09106 (15)	0.0645 (10)	
H1A	0.1300	0.4680	−0.1057	0.077*	
N2	0.3298 (2)	0.5438 (4)	−0.09270 (19)	0.0652 (10)	
C1	0.1136 (4)	0.3372 (6)	0.0604 (2)	0.111	
H1B	0.1518	0.3704	0.0982	0.167*	
H1C	0.1098	0.2352	0.0611	0.167*	
H1D	0.0553	0.3773	0.0528	0.167*	
C2	0.1514 (5)	0.3823 (7)	0.0116 (3)	0.126 (2)	
H2A	0.1543	0.4855	0.0113	0.151*	
H2B	0.2119	0.3470	0.0216	0.151*	
C3	0.1044 (4)	0.3362 (6)	−0.0463 (2)	0.0873 (16)	
H3A	0.0437	0.3706	−0.0567	0.105*	
H3B	0.1024	0.2330	−0.0467	0.105*	
C4	0.1965 (3)	0.3030 (4)	−0.11057 (18)	0.0605 (11)	
C5	0.2291 (3)	0.3690 (4)	−0.15806 (17)	0.0522 (10)	
C6	0.1972 (3)	0.3081 (5)	−0.2145 (2)	0.0753 (13)	
H6A	0.1583	0.2313	−0.2207	0.090*	
C7	0.2226 (4)	0.3604 (6)	−0.2617 (2)	0.0874 (16)	
H7A	0.2008	0.3191	−0.2993	0.105*	
C8	0.2800 (3)	0.4730 (6)	−0.2526 (2)	0.0814 (14)	
H8A	0.2968	0.5083	−0.2842	0.098*	
C9	0.3126 (3)	0.5336 (5)	−0.1981 (2)	0.0650 (12)	
H9A	0.3516	0.6103	−0.1923	0.078*	
C10	0.2881 (3)	0.4819 (4)	−0.15181 (17)	0.0507 (10)	
O4	0.1360 (2)	−0.3230 (3)	−0.12406 (13)	0.0808 (10)	
O5	−0.0495 (3)	0.1095 (5)	−0.1661 (2)	0.1416 (18)	
O6	−0.0153 (2)	−0.0953 (5)	−0.12712 (17)	0.1127 (14)	
N3	0.1889 (2)	−0.1148 (3)	−0.07927 (14)	0.0598 (9)	
H3C	0.1982	−0.0265	−0.0850	0.072*	
N4	−0.0107 (3)	−0.0024 (5)	−0.16209 (18)	0.0771 (11)	
C11	0.1902 (4)	−0.1746 (6)	0.0794 (2)	0.115	
H11A	0.1509	−0.1381	0.0997	0.173*	
H11B	0.2500	−0.1450	0.1001	0.173*	
H11C	0.1873	−0.2767	0.0785	0.173*	
C12	0.1632 (4)	−0.1196 (6)	0.0183 (2)	0.106	
H12A	0.1651	−0.0164	0.0198	0.127*	
H12B	0.1020	−0.1475	−0.0017	0.127*	
C13	0.2203 (3)	−0.1708 (4)	−0.01861 (18)	0.0720 (13)	
H13A	0.2813	−0.1409	0.0003	0.086*	
H13B	0.2193	−0.2741	−0.0200	0.086*	
C14	0.1471 (3)	−0.1932 (4)	−0.12538 (18)	0.0560 (11)	
C15	0.1150 (3)	−0.1159 (4)	−0.18493 (18)	0.0531 (10)	
C16	0.1612 (3)	−0.1351 (5)	−0.2252 (2)	0.0692 (12)	
H16A	0.2118	−0.1919	−0.2148	0.083*	
C17	0.1330 (3)	−0.0707 (5)	−0.2807 (2)	0.0776 (14)	
H17A	0.1650	−0.0847	−0.3072	0.093*	
C18	0.0584 (3)	0.0135 (5)	−0.29718 (19)	0.0671 (12)	
H18A	0.0392	0.0549	−0.3348	0.081*	
C19	0.0128 (3)	0.0357 (4)	−0.25787 (18)	0.0597 (11)	
H19A	−0.0374	0.0936	−0.2682	0.072*	
C20	0.0415 (3)	−0.0284 (4)	−0.20266 (17)	0.0517 (10)	
O7	0.4897 (2)	0.9114 (3)	0.26431 (12)	0.0691 (9)	
O8	0.3181 (3)	0.5045 (5)	0.14397 (19)	0.1227 (15)	
O9	0.3425 (2)	0.6663 (4)	0.21211 (18)	0.0980 (12)	
N5	0.5255 (2)	0.6835 (3)	0.29072 (13)	0.0589 (9)	
H5A	0.5387	0.6018	0.2793	0.071*	
N6	0.3587 (3)	0.6078 (5)	0.1705 (2)	0.0788 (11)	
C21	0.5982 (4)	0.6457 (6)	0.45860 (19)	0.111 (2)	
H21A	0.6482	0.5978	0.4858	0.167*	
H21B	0.5440	0.6041	0.4610	0.167*	
H21C	0.5999	0.7448	0.4689	0.167*	
C22	0.6022 (4)	0.6307 (6)	0.3953 (2)	0.0946 (17)	
H22A	0.6577	0.6704	0.3933	0.114*	
H22B	0.6010	0.5305	0.3851	0.114*	
C23	0.5289 (3)	0.7021 (5)	0.35266 (18)	0.0764 (14)	
H23A	0.5327	0.8030	0.3618	0.092*	
H23B	0.4738	0.6670	0.3572	0.092*	
C24	0.5028 (3)	0.7884 (4)	0.25144 (18)	0.0520 (10)	
C25	0.4954 (3)	0.7494 (4)	0.18769 (16)	0.0472 (9)	
C26	0.4311 (3)	0.6667 (4)	0.15025 (18)	0.0594 (11)	
C27	0.4272 (4)	0.6379 (5)	0.0922 (2)	0.0803 (15)	
H27A	0.3831	0.5791	0.0682	0.096*	
C28	0.4895 (4)	0.6978 (7)	0.0712 (2)	0.0895 (17)	
H28A	0.4872	0.6816	0.0319	0.107*	
C29	0.5551 (4)	0.7811 (6)	0.1067 (2)	0.0786 (15)	
H29A	0.5978	0.8203	0.0919	0.094*	
C30	0.5581 (3)	0.8073 (4)	0.16499 (19)	0.0632 (12)	
H30A	0.6029	0.8647	0.1892	0.076*	

### Atomic displacement parameters (Å^2^)

**Table:**

	*U*^11^	*U*^22^	*U*^33^	*U*^12^	*U*^13^	*U*^23^
O1	0.122 (3)	0.0300 (15)	0.095 (2)	−0.0039 (17)	0.039 (2)	0.0083 (15)
O2	0.139 (3)	0.084 (3)	0.121 (3)	−0.061 (2)	0.064 (3)	−0.040 (2)
O3	0.094 (2)	0.079 (2)	0.061 (2)	−0.0115 (19)	0.0156 (18)	−0.0069 (18)
N1	0.085 (3)	0.046 (2)	0.073 (2)	−0.0003 (19)	0.039 (2)	0.0162 (18)
N2	0.068 (2)	0.051 (2)	0.084 (3)	−0.011 (2)	0.034 (2)	−0.016 (2)
C1	0.111	0.111	0.111	0.000	0.035	0.000
C2	0.193 (7)	0.117 (5)	0.093 (4)	−0.035 (5)	0.080 (5)	0.006 (4)
C3	0.108 (4)	0.083 (4)	0.082 (4)	−0.011 (3)	0.047 (3)	0.014 (3)
C4	0.082 (3)	0.036 (2)	0.062 (3)	−0.008 (2)	0.021 (2)	0.003 (2)
C5	0.063 (3)	0.038 (2)	0.056 (2)	0.002 (2)	0.021 (2)	−0.001 (2)
C6	0.086 (3)	0.065 (3)	0.071 (3)	−0.015 (3)	0.020 (3)	−0.014 (3)
C7	0.092 (4)	0.111 (4)	0.056 (3)	−0.005 (4)	0.019 (3)	−0.021 (3)
C8	0.087 (4)	0.099 (4)	0.069 (3)	0.005 (3)	0.039 (3)	0.009 (3)
C9	0.068 (3)	0.057 (3)	0.080 (3)	−0.003 (2)	0.036 (3)	0.006 (2)
C10	0.055 (2)	0.043 (2)	0.058 (3)	−0.001 (2)	0.023 (2)	−0.005 (2)
O4	0.110 (3)	0.0349 (16)	0.082 (2)	−0.0110 (17)	0.0087 (19)	0.0087 (15)
O5	0.171 (4)	0.142 (4)	0.147 (4)	0.089 (4)	0.100 (3)	0.042 (3)
O6	0.099 (3)	0.145 (4)	0.116 (3)	0.027 (3)	0.064 (2)	0.058 (3)
N3	0.081 (3)	0.0338 (17)	0.059 (2)	−0.0053 (18)	0.0145 (19)	0.0063 (17)
N4	0.069 (3)	0.088 (3)	0.077 (3)	0.015 (2)	0.028 (2)	0.016 (3)
C11	0.115	0.115	0.115	0.000	0.036	0.000
C12	0.105	0.105	0.105	0.000	0.032	0.000
C13	0.098 (4)	0.050 (3)	0.065 (3)	−0.003 (3)	0.021 (3)	0.004 (2)
C14	0.065 (3)	0.037 (2)	0.063 (3)	−0.002 (2)	0.016 (2)	0.006 (2)
C15	0.057 (3)	0.034 (2)	0.067 (3)	0.000 (2)	0.019 (2)	0.000 (2)
C16	0.069 (3)	0.061 (3)	0.081 (3)	0.017 (2)	0.028 (3)	0.000 (3)
C17	0.089 (4)	0.084 (3)	0.073 (3)	0.001 (3)	0.045 (3)	−0.004 (3)
C18	0.075 (3)	0.072 (3)	0.054 (3)	−0.007 (3)	0.019 (2)	0.006 (2)
C19	0.055 (3)	0.058 (3)	0.061 (3)	0.002 (2)	0.012 (2)	0.014 (2)
C20	0.057 (3)	0.044 (2)	0.058 (3)	−0.006 (2)	0.023 (2)	0.002 (2)
O7	0.107 (2)	0.0359 (15)	0.073 (2)	0.0053 (16)	0.0406 (18)	−0.0046 (14)
O8	0.108 (3)	0.116 (3)	0.138 (4)	−0.048 (3)	0.029 (3)	−0.039 (3)
O9	0.083 (2)	0.111 (3)	0.114 (3)	−0.024 (2)	0.052 (2)	−0.029 (2)
N5	0.099 (3)	0.0348 (17)	0.050 (2)	0.0123 (18)	0.0333 (19)	0.0000 (16)
N6	0.069 (3)	0.075 (3)	0.088 (3)	−0.003 (2)	0.018 (2)	−0.007 (2)
C21	0.165 (6)	0.107 (4)	0.051 (3)	0.029 (4)	0.018 (3)	−0.005 (3)
C22	0.114 (4)	0.094 (4)	0.062 (3)	0.027 (3)	0.007 (3)	−0.010 (3)
C23	0.119 (4)	0.055 (3)	0.060 (3)	0.018 (3)	0.035 (3)	0.001 (2)
C24	0.060 (2)	0.044 (2)	0.056 (2)	−0.003 (2)	0.024 (2)	0.000 (2)
C25	0.057 (3)	0.039 (2)	0.050 (2)	0.012 (2)	0.022 (2)	0.0051 (19)
C26	0.062 (3)	0.059 (3)	0.055 (3)	0.006 (2)	0.017 (2)	0.000 (2)
C27	0.094 (4)	0.084 (4)	0.058 (3)	0.019 (3)	0.016 (3)	−0.008 (3)
C28	0.099 (4)	0.111 (5)	0.060 (3)	0.033 (4)	0.026 (3)	0.009 (3)
C29	0.086 (4)	0.088 (4)	0.076 (3)	0.031 (3)	0.046 (3)	0.034 (3)
C30	0.074 (3)	0.053 (3)	0.068 (3)	0.007 (2)	0.030 (2)	0.013 (2)

### Geometric parameters (Å, °)

**Table:**

O1—C4	1.228 (4)	C13—H13A	0.9700
O2—N2	1.223 (4)	C13—H13B	0.9700
O3—N2	1.214 (4)	C14—C15	1.521 (5)
N1—C4	1.328 (5)	C15—C20	1.377 (5)
N1—C3	1.444 (5)	C15—C16	1.382 (5)
N1—H1A	0.8600	C16—C17	1.384 (6)
N2—C10	1.464 (5)	C16—H16A	0.9300
C1—C2	1.518 (7)	C17—C18	1.372 (6)
C1—H1B	0.9600	C17—H17A	0.9300
C1—H1C	0.9600	C18—C19	1.360 (5)
C1—H1D	0.9600	C18—H18A	0.9300
C2—C3	1.404 (7)	C19—C20	1.377 (5)
C2—H2A	0.9700	C19—H19A	0.9300
C2—H2B	0.9700	O7—C24	1.228 (4)
C3—H3A	0.9700	O8—N6	1.222 (5)
C3—H3B	0.9700	O9—N6	1.221 (5)
C4—C5	1.508 (5)	N5—C24	1.324 (5)
C5—C10	1.391 (5)	N5—C23	1.458 (5)
C5—C6	1.391 (5)	N5—H5A	0.8600
C6—C7	1.389 (6)	N6—C26	1.482 (6)
C6—H6A	0.9300	C21—C22	1.524 (6)
C7—C8	1.365 (7)	C21—H21A	0.9600
C7—H7A	0.9300	C21—H21B	0.9600
C8—C9	1.352 (6)	C21—H21C	0.9600
C8—H8A	0.9300	C22—C23	1.440 (6)
C9—C10	1.359 (5)	C22—H22A	0.9700
C9—H9A	0.9300	C22—H22B	0.9700
O4—C14	1.234 (4)	C23—H23A	0.9700
O5—N4	1.206 (5)	C23—H23B	0.9700
O6—N4	1.219 (5)	C24—C25	1.518 (5)
N3—C14	1.309 (5)	C25—C26	1.362 (5)
N3—C13	1.459 (5)	C25—C30	1.380 (5)
N3—H3C	0.8600	C26—C27	1.379 (6)
N4—C20	1.470 (5)	C27—C28	1.358 (7)
C11—C12	1.464 (7)	C27—H27A	0.9300
C11—H11A	0.9600	C28—C29	1.358 (7)
C11—H11B	0.9600	C28—H28A	0.9300
C11—H11C	0.9600	C29—C30	1.385 (6)
C12—C13	1.518 (6)	C29—H29A	0.9300
C12—H12A	0.9700	C30—H30A	0.9300
C12—H12B	0.9700		
			
C4—N1—C3	122.6 (4)	H13A—C13—H13B	108.0
C4—N1—H1A	118.7	O4—C14—N3	125.0 (4)
C3—N1—H1A	118.7	O4—C14—C15	119.1 (4)
O3—N2—O2	123.7 (4)	N3—C14—C15	115.8 (3)
O3—N2—C10	119.4 (4)	C20—C15—C16	116.6 (4)
O2—N2—C10	116.9 (4)	C20—C15—C14	124.8 (4)
C2—C1—H1B	109.5	C16—C15—C14	118.6 (4)
C2—C1—H1C	109.5	C15—C16—C17	120.8 (4)
H1B—C1—H1C	109.5	C15—C16—H16A	119.6
C2—C1—H1D	109.5	C17—C16—H16A	119.6
H1B—C1—H1D	109.5	C18—C17—C16	120.8 (4)
H1C—C1—H1D	109.5	C18—C17—H17A	119.6
C3—C2—C1	116.0 (5)	C16—C17—H17A	119.6
C3—C2—H2A	108.3	C19—C18—C17	119.3 (4)
C1—C2—H2A	108.3	C19—C18—H18A	120.4
C3—C2—H2B	108.3	C17—C18—H18A	120.4
C1—C2—H2B	108.3	C18—C19—C20	119.5 (4)
H2A—C2—H2B	107.4	C18—C19—H19A	120.3
C2—C3—N1	113.6 (4)	C20—C19—H19A	120.3
C2—C3—H3A	108.8	C19—C20—C15	123.0 (4)
N1—C3—H3A	108.8	C19—C20—N4	117.3 (4)
C2—C3—H3B	108.8	C15—C20—N4	119.7 (4)
N1—C3—H3B	108.8	C24—N5—C23	122.0 (3)
H3A—C3—H3B	107.7	C24—N5—H5A	119.0
O1—C4—N1	124.7 (4)	C23—N5—H5A	119.0
O1—C4—C5	119.6 (4)	O9—N6—O8	124.1 (5)
N1—C4—C5	115.5 (3)	O9—N6—C26	118.1 (4)
C10—C5—C6	116.4 (4)	O8—N6—C26	117.8 (5)
C10—C5—C4	127.6 (4)	C22—C21—H21A	109.5
C6—C5—C4	116.0 (4)	C22—C21—H21B	109.5
C7—C6—C5	120.9 (4)	H21A—C21—H21B	109.5
C7—C6—H6A	119.5	C22—C21—H21C	109.5
C5—C6—H6A	119.5	H21A—C21—H21C	109.5
C8—C7—C6	119.6 (5)	H21B—C21—H21C	109.5
C8—C7—H7A	120.2	C23—C22—C21	111.7 (4)
C6—C7—H7A	120.2	C23—C22—H22A	109.3
C9—C8—C7	120.8 (5)	C21—C22—H22A	109.3
C9—C8—H8A	119.6	C23—C22—H22B	109.3
C7—C8—H8A	119.6	C21—C22—H22B	109.3
C8—C9—C10	119.6 (4)	H22A—C22—H22B	107.9
C8—C9—H9A	120.2	C22—C23—N5	114.4 (4)
C10—C9—H9A	120.2	C22—C23—H23A	108.7
C9—C10—C5	122.6 (4)	N5—C23—H23A	108.7
C9—C10—N2	117.9 (4)	C22—C23—H23B	108.7
C5—C10—N2	119.4 (4)	N5—C23—H23B	108.7
C14—N3—C13	122.8 (3)	H23A—C23—H23B	107.6
C14—N3—H3C	118.6	O7—C24—N5	123.8 (4)
C13—N3—H3C	118.6	O7—C24—C25	120.4 (4)
O5—N4—O6	122.6 (5)	N5—C24—C25	115.7 (3)
O5—N4—C20	118.1 (4)	C26—C25—C30	117.0 (4)
O6—N4—C20	119.3 (4)	C26—C25—C24	126.2 (4)
C12—C11—H11A	109.5	C30—C25—C24	116.7 (4)
C12—C11—H11B	109.5	C25—C26—C27	123.2 (4)
H11A—C11—H11B	109.5	C25—C26—N6	119.9 (4)
C12—C11—H11C	109.5	C27—C26—N6	116.8 (4)
H11A—C11—H11C	109.5	C28—C27—C26	118.1 (5)
H11B—C11—H11C	109.5	C28—C27—H27A	121.0
C11—C12—C13	114.4 (5)	C26—C27—H27A	121.0
C11—C12—H12A	108.7	C27—C28—C29	121.1 (5)
C13—C12—H12A	108.7	C27—C28—H28A	119.5
C11—C12—H12B	108.7	C29—C28—H28A	119.5
C13—C12—H12B	108.7	C28—C29—C30	119.7 (5)
H12A—C12—H12B	107.6	C28—C29—H29A	120.1
N3—C13—C12	111.7 (4)	C30—C29—H29A	120.1
N3—C13—H13A	109.3	C25—C30—C29	120.8 (4)
C12—C13—H13A	109.3	C25—C30—H30A	119.6
N3—C13—H13B	109.3	C29—C30—H30A	119.6
C12—C13—H13B	109.3		
			
C1—C2—C3—N1	179.2 (5)	C17—C18—C19—C20	−1.0 (6)
C4—N1—C3—C2	97.2 (6)	C18—C19—C20—C15	−0.3 (6)
C3—N1—C4—O1	3.3 (7)	C18—C19—C20—N4	−179.3 (4)
C3—N1—C4—C5	179.1 (4)	C16—C15—C20—C19	1.3 (6)
O1—C4—C5—C10	−114.7 (5)	C14—C15—C20—C19	−177.6 (4)
N1—C4—C5—C10	69.3 (6)	C16—C15—C20—N4	−179.7 (4)
O1—C4—C5—C6	64.3 (6)	C14—C15—C20—N4	1.3 (6)
N1—C4—C5—C6	−111.7 (4)	O5—N4—C20—C19	−29.9 (6)
C10—C5—C6—C7	−1.0 (6)	O6—N4—C20—C19	148.1 (4)
C4—C5—C6—C7	179.9 (4)	O5—N4—C20—C15	151.1 (5)
C5—C6—C7—C8	0.2 (8)	O6—N4—C20—C15	−31.0 (6)
C6—C7—C8—C9	0.3 (8)	C21—C22—C23—N5	−176.0 (4)
C7—C8—C9—C10	0.1 (7)	C24—N5—C23—C22	−141.0 (5)
C8—C9—C10—C5	−0.9 (7)	C23—N5—C24—O7	6.1 (7)
C8—C9—C10—N2	175.7 (4)	C23—N5—C24—C25	−175.4 (4)
C6—C5—C10—C9	1.4 (6)	O7—C24—C25—C26	−110.9 (5)
C4—C5—C10—C9	−179.6 (4)	N5—C24—C25—C26	70.5 (5)
C6—C5—C10—N2	−175.2 (4)	O7—C24—C25—C30	66.7 (5)
C4—C5—C10—N2	3.9 (6)	N5—C24—C25—C30	−111.9 (4)
O3—N2—C10—C9	−158.9 (4)	C30—C25—C26—C27	0.9 (6)
O2—N2—C10—C9	23.5 (6)	C24—C25—C26—C27	178.5 (4)
O3—N2—C10—C5	17.8 (6)	C30—C25—C26—N6	−176.6 (4)
O2—N2—C10—C5	−159.8 (4)	C24—C25—C26—N6	1.1 (6)
C14—N3—C13—C12	−106.3 (5)	O9—N6—C26—C25	21.4 (6)
C11—C12—C13—N3	178.8 (4)	O8—N6—C26—C25	−159.5 (4)
C13—N3—C14—O4	−6.3 (7)	O9—N6—C26—C27	−156.2 (4)
C13—N3—C14—C15	177.1 (4)	O8—N6—C26—C27	22.9 (6)
O4—C14—C15—C20	106.6 (5)	C25—C26—C27—C28	−1.5 (7)
N3—C14—C15—C20	−76.5 (5)	N6—C26—C27—C28	176.0 (4)
O4—C14—C15—C16	−72.3 (5)	C26—C27—C28—C29	1.5 (8)
N3—C14—C15—C16	104.6 (5)	C27—C28—C29—C30	−0.9 (8)
C20—C15—C16—C17	−1.1 (6)	C26—C25—C30—C29	−0.2 (6)
C14—C15—C16—C17	177.9 (4)	C24—C25—C30—C29	−178.1 (4)
C15—C16—C17—C18	−0.1 (7)	C28—C29—C30—C25	0.2 (7)
C16—C17—C18—C19	1.2 (7)		

### Hydrogen-bond geometry (Å, °)

**Table:**

*D*—H···*A*	*D*—H	H···*A*	*D*···*A*	*D*—H···*A*
N1—H1A···O4^i^	0.86	2.02	2.854 (5)	163
N3—H3C···O1	0.86	1.98	2.840 (4)	177
N5—H5A···O7^ii^	0.86	2.04	2.843 (4)	154
C6—H6A···Cg2	0.93	2.86	3.751 (5)	162

Symmetry codes: (i) *x*, *y*+1, *z*; (ii) −*x*+1, *y*−1/2, −*z*+1/2.
